# Alcoholic Liver Disease/Nonalcoholic Fatty Liver Disease Index at Diagnosis Is Associated with All-Cause Mortality during Follow-Up in Patients with Antineutrophil Cytoplasmic Antibody-Associated Vasculitis

**DOI:** 10.3390/medicina60030381

**Published:** 2024-02-24

**Authors:** Minsuk Cho, Woongchan Rah, Jason Jungsik Song, Yong-Beom Park, Sang-Won Lee

**Affiliations:** 1 Department of Medicine, Yonsei University College of Medicine, Seoul 03722, Republic of Korea; 2 Division of Rheumatology, Department of Internal Medicine, Yonsei University College of Medicine, Seoul 03722, Republic of Korea; 3 Institute for Immunology and Immunological Diseases, Yonsei University College of Medicine, Seoul 03722, Republic of Korea

**Keywords:** alcoholic liver disease/nonalcoholic fatty liver disease index, antineutrophil cytoplasmic antibody, vasculitis, estimate, mortality

## Abstract

*Background and Objectives*: The purpose of this study was to investigate whether a new index related to chronic liver disease, the alcoholic liver disease/nonalcoholic fatty liver disease index (ANI) at diagnosis, is associated with all-cause mortality during follow-up in patients with antineutrophil cytoplasmic antibody-associated vasculitis (AAV). *Materials and Methods*: In this study, we included 270 patients with AAV. ANI was calculated using the following equation: ANI = −58.5 + 0.637 (adjusted mean corpuscular volume) + 3.91 (adjusted aspartate transaminase/alanine transaminase) − 0.406 (body mass index) + 6.35 (if male sex). All-cause mortality was defined as death from any cause during follow-up. *Results*: The median age of the 270 patients with AAV was 61.0 years (34.4% male and 66.6% female). The median ANI was significantly higher in deceased patients than in surviving patients. In the receiver operating characteristic curve analysis, ANI at diagnosis exhibited a statistically significant area under the curve for all-cause mortality during follow-up, and its cut-off was determined to be −0.59. Patients with ANI at diagnosis ≥ −0.59 exhibited a significantly higher risk for all-cause mortality and a significantly lower cumulative patient survival rate than those without. In the multivariable Cox analysis, ANI at diagnosis ≥ −0.59, together with age at diagnosis, was independently associated with all-cause mortality. *Conclusions*: This study is the first to demonstrate the predictive potential of ANI at diagnosis for all-cause mortality during follow-up in AAV patients without significant chronic liver diseases.

## 1. Introduction

Alcoholic liver disease (ALD) is a major chronic disease that can provoke simple fatty liver disease, alcoholic steatohepatitis, liver cirrhosis, and hepatocellular carcinoma [1]. Nonalcoholic fatty liver disease (NAFLD) is the most common chronic liver disease and is mainly caused by obesity and insulin resistance [2]. NAFLD may progress to liver cirrhosis or hepatocellular carcinoma; however, its probability is lower than that of ALD [3]. To differentiate between ALD and NAFLD, liver biopsy is the gold standard diagnostic modality, but it is not strongly recommended because of an invasive procedure [4]. However, Dunn et al. proposed a new index, the ALD/NAFLD index (ANI), for discriminating the two diseases. ANI is composed of four parameters including the mean corpuscular volume (MCV), aspartate aminotransferase (AST)/alanine aminotransferase (ALT) ratio, body mass index (BMI), and male sex [5].

Antineutrophil cytoplasmic antibody (ANCA)-associated vasculitis (AAV) is characterised by an association with ANCA and typical findings of necrotic fibrosis and inflammation in small-sized vessels [6,7]. Based on clinical, laboratory, radiological, and histological features, AAV is categorised into three subtypes: microscopic polyangiitis (MPA), granulomatosis with polyangiitis (GPA), and eosinophilic GPA (EGPA) [8,9,10]. AAV can affect almost all the major organs; however, according to the classification criteria for AAV, the Birmingham Vasculitis Activity Score (BVAS) and the Five-Factor Score (FFS), the liver is not considered to be the major organ that is mainly affected [11,12,13]. Nevertheless, the indirect link between the alterations in hepatic structure and function and poor outcomes in patients with AAV has been introduced. In terms of NAFLD, several previous studies reported that the indices for estimating liver fibrosis or fatty liver disease could estimate all-cause mortality in patients with AAV [14,15,16]. However, in terms of ALD, no research on their relationships has been reported yet. Although, among the parameters of ANI, the contribution of AST and male sex at diagnosis to the estimation of all-cause mortality was demonstrated in patients with systemic vasculitis to date [17,18]. Therefore, although ANI was developed as an index for distinguishing ALD from NAFLD, it could be reasonably speculated that ANI could be used to estimate poor outcomes during follow-up in patients with AAV. Hence, in the present study, we aimed to investigate whether ANI at diagnosis, a new index related to chronic liver disease, is associated with all-cause mortality during follow-up in patients with AAV.

## 2. Materials and Methods

### 2.1. Patients

**Figure 1:**
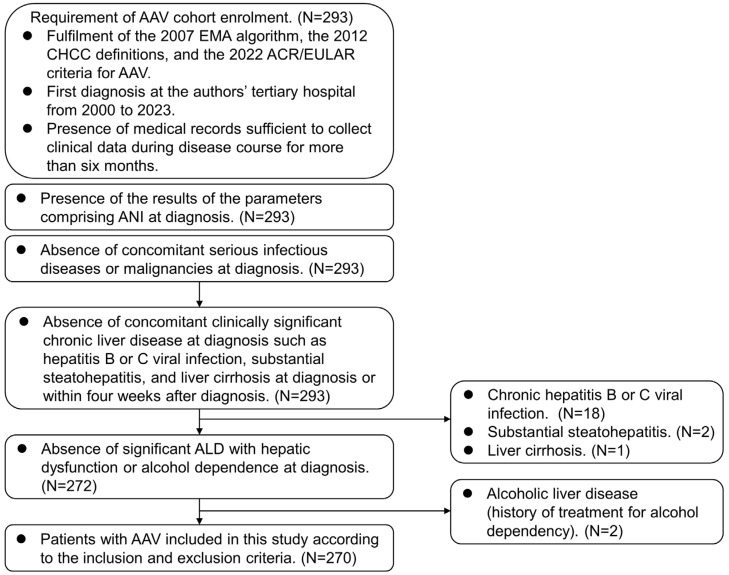
Algorithm for patient selection. AAV: ANCA-associated vasculitis; ANCA: antineutrophil cytoplasmic antibody; EMA: European Medicine Agency; CHCC: Chapel Hill Consensus Conference; ACR: American College of Rheumatology; EULAR: European Alliance of Associations for Rheumatology; ANI: the alcoholic liver disease/nonalcoholic fatty liver disease index: ALD: alcoholic liver disease.

The present study screened 293 patients with AAV and included 270 according to the inclusion and exclusion criteria. The inclusion criteria were (i) patients who fulfilled the 2007 European Medicine Agency algorithm for AAV, the 2012 revised International Chapel Hill Consensus Conference Nomenclature of Vasculitides, and the 2022 American College of Rheumatology/European Alliance of Associations for Rheumatology classification criteria for AAV [6,7,8,9,10]; (ii) patients who were first diagnosed with AAV in the authors’ tertiary care university hospital from 2000 to 2023; (iii) patients who had medical records sufficiently well-documented to collect clinical, laboratory, radiological, and histological data not only at diagnosis but also during follow-up; (iv) patients who had results of the parameters comprising ANI at diagnosis; and (v) patients who had been followed up for more than 6 months. The exclusion criteria were (i) patients who had concurrent serious infectious diseases or malignancies screened by neck, chest, and abdomen–pelvis computed tomography (CT) scans, endoscopies, and/or a fluorodeoxyglucose (FDG)-positron emission tomography (PET) scan at diagnosis; (ii) patients who had a concomitant clinically significant chronic liver disease at diagnosis such as hepatitis B or C viral infection confirmed by tests for HBsAg, anti-HBs, anti-HCV, and/or anti-HBc, substantial steatohepatitis and liver cirrhosis determined by abdominal ultrasonography with transient elastography (Fibroscan^®^, EchoSens, Paris, France), and abdomen CT scans at diagnosis or within four weeks after diagnosis; and (iii) patients who had significant ALD with hepatic dysfunction or alcohol dependence at diagnosis, which were identified by the 10th revised International Classification Diseases—10 and the Korean Drug Utilization Review system at diagnosis. Of the 293 patients, 18, 2, and 1 were excluded owing to chronic hepatitis B or C viral infection, substantial steatohepatitis, and significant liver cirrhosis. Of the 272 patients, 2 were further excluded owing to alcoholic liver disease with a history of treatment for alcohol dependency, and finally, 270 were included and analysed in this study (Figure 1).

### 2.2. Ethical Approval

The present study was approved by the Institutional Review Board (IRB) of Severance Hospital (Seoul, Republic of Korea, IRB No. 4-2020-1071), and conducted in accordance with the Declaration of Helsinki. Given the retrospective design of this study and the use of anonymised patient data, requirements for informed written consent were waived. 

### 2.3. Equation for ANI

ANI was calculated using the following equation: ANI = −58.5 + 0.637 (adjusted MCV) + 3.91 (adjusted AST/ALT) − 0.406 (BMI) + 6.35 (if male sex). In terms of the adjusted MCV, the range of MCV as a variable for ALI is 92–103 fL. Therefore, an MCV < 92 fL was set to 92 fL, and an MCV > 103 fL was set to 103 fL. In terms of the adjusted AST/ALT ratio, the range of AST/ALT as a parameter of ALI cannot exceed 3. Therefore, AST/ALT above 3 was set at 3 [5].

### 2.4. Data at AAV Diagnosis

Demographic data at diagnosis included age, male sex, BMI, and smoking history. AAV subtype, ANCA type, and positivity, BVAS, and FFS at diagnosis were collected [12,13]. Myeloperoxidase (MPO)-ANCA, and proteinase 3 (PR3)-ANCA were measured using an immunoassay, whereas perinuclear (P)-ANCA and cytoplasmic (C)-ANCA were detected using an indirect immunofluorescence assay. All four ANCA types have been accepted as ANCA positivity according to the 2022 new criteria for AAV [8,9,10]. Type 2 diabetes mellitus, arterial hypertension, and dyslipidaemia at diagnosis were identified as comorbidities. Laboratory data at diagnosis, including the erythrocyte sedimentation rate (ESR), C-reactive protein (CRP), MCV, and AST/ALT were collected.

### 2.5. Data during Follow-Up

All-cause mortality was defined as death from any cause. Apart from the follow-up duration, the follow-up duration based on all-cause mortality was used in the statistical analyses. Among deceased patients, the follow-up duration based on all-cause mortality was defined as the period from AAV diagnosis to death. Whereas, among surviving patients, it was defined as that from AAV diagnosis to the last visit. Additionally, glucocorticoids and immunosuppressive drugs administered during follow-up were also recorded.

### 2.6. Statistical Analyses

Statistical analyses were performed using SPSS Statistics version 26 (IBM Corporation, Armonk, NY, USA) for Windows 10 (Microsoft Corporation, Redmond, WA, USA). Categorical and continuous variables are expressed as number (percentage) and median (25th and 75th percentiles), respectively. The Mann–Whitney U test was used to compare significant differences between two continuous variables. The optimal ANI cut-off for all-cause mortality was extrapolated by performing the receiver operating characteristic (ROC) curve analysis, and the value with the maximum sum of sensitivity and specificity was selected. The relative risk (RR) for the cut-off of ANI for all-cause mortality was analysed using contingency tables and the chi-square test. The comparison of the cumulative survival rates between the two groups was analysed by the Kaplan–Meier survival analysis with the log-rank test. The multivariable Cox hazard model using variables with statistical significance in the univariable Cox hazard model was conducted to appropriately obtain the hazard ratios (HRs) during the considerable follow-up duration. A *p*-value of less than 0.05 was considered statistically significant.

## 3. Results

### 3.1. Characteristics

Regarding the variables at diagnosis, the median age of the 270 patients was 61.0 years. Among the 270 patients, 93 (34.4%) and 177 (66.6%) were men and women, and further, 152, 66, and 52 were diagnosed with MPA, GPA, and EGPA, respectively. The median BMI was 22.4 kg/m^2^, and 3.3% of the patients had a history of smoking. MPO-ANCA (or P-ANCA) and PR3-ANCA (or C-ANCA) were detected in 191 and 41 patients, respectively. The median BVAS, FFS, ESR, and CRP were 12.0, 1.0, 60.5 mm/h, and 13.8 mg/L, respectively. Overall, 72, 110, and 55 patients had type 2 diabetes mellitus, arterial hypertension, and dyslipidaemia, respectively. The median adjusted MCV, the adjusted AST/ALT ratio, and ANI were 92.0 fL, 1.2, and −1.15, respectively. Regarding the variables during follow-up, 32 (11.9%) patients died during the median follow-up of 47.8 months based on all-cause mortality. Glucocorticoids were administered to 254 (94.1%) patients, and the most frequently administered immunosuppressive drug was cyclophosphamide (56.7%) followed by azathioprine (54.1%) (Table 1).

### 3.2. Comparison of ANI at Diagnosis between Surviving and Deceased Patients

**Figure 2:**
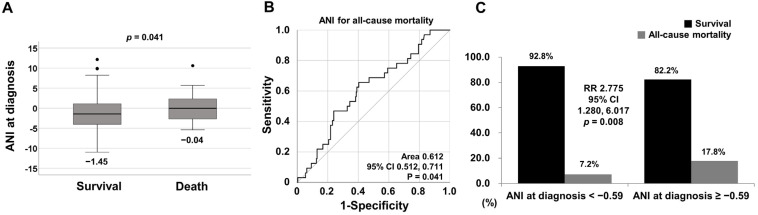
(**A**) Comparison of ANI at diagnosis between surviving and deceased patients, (**B**) the obtained optimal cut-off of ANI for all-cause mortality, and (**C**) the relative risk for ANI at diagnosis ≥ −0.59 for all-cause mortality. ANI: the alcoholic liver disease/nonalcoholic fatty liver disease index; RR: relative risk; CI: confidence interval.

Deceased patients exhibited a significantly higher median ANI at diagnosis than surviving patients (−0.04 vs. −1.45, *p* = 0.041) (Figure 2A).

### 3.3. Optimal Cut-off of ANI for All-Cause Mortality

The ROC curve analysis of ANI at diagnosis for all-cause mortality during follow-up demonstrated a statistically significant area under the curve (area 0.612, 95% confidence interval [CI] 0.512, 0.711). When the cut-off of ANI at diagnosis for all-cause mortality was set as −0.59, the sensitivity and specificity were 65.6% and 59.7%, respectively (Figure 2B).

### 3.4. Relative Risk of ANI at Diagnosis ≥ −0.59 for All-Cause Mortality

When we divided patients into two groups based on ANI at diagnosis ≥ −0.59, 118 of the 270 patients were assigned to the group with ANI at diagnosis ≥ −0.59. All-cause mortality was identified more frequently in patients with ANI at diagnosis ≥ −0.59 than those with ANI at diagnosis < −0.59 (17.8% vs 7.2%, *p* = 0.008). Furthermore, patients with ANI at diagnosis ≥ −0.59 exhibited a significantly higher risk of all-cause mortality than those with ANI at diagnosis < −0.59 (RR 2.775, 95% CI 1.280, 6.017) (Figure 2C).

### 3.5. Comparison of Cumulative Patient Survival Rates

**Figure 3:**
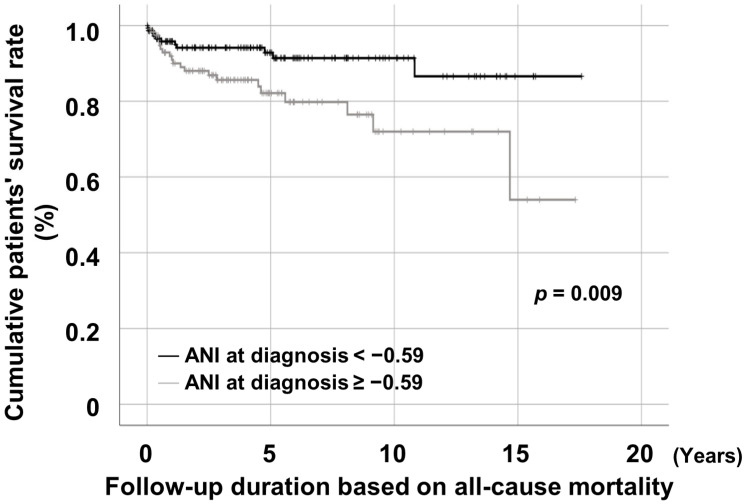
Comparison of cumulative patient survival rates between patients with ANI at diagnosis ≥ −0.59 and those without. ANI: the alcoholic liver disease/nonalcoholic fatty liver disease index.

Patients with ANI at diagnosis ≥ −0.59 exhibited a significantly lower cumulative patient survival rate than those with ANI at diagnosis < −0.59 (Figure 3).

### 3.6. Cox Hazards Model Analysis for All-Cause Mortality

In the univariable Cox analysis with variables at diagnosis, age (HR 1.089), BVAS (HR 1.086), FFS (HR 2.019), ESR (HR 1.012), CRP (HR 1.009), and ANI at diagnosis ≥ −0.59 (HR 2.550) were significantly associated with all-cause mortality. In the multivariable Cox analysis, both age (HR 1.061, 95% CI 1.017, 1.107) and ANI at diagnosis ≥ −0.59 (HR 2.479, 95% CI 1.149, 5.349) were independently associated with all-cause mortality (Table 2).

## 4. Discussion

In the present study, we investigated the potential of ANI at diagnosis to estimate all-cause mortality during follow-up in patients with AAV and obtained several interesting results. In the comparative analysis, the median ANI at diagnosis in deceased patients was significantly higher than that in surviving patients. The ROC curve analysis revealed that ANI at diagnosis exhibited a statistically significant area under the curve for all-cause mortality during follow-up. Furthermore, the cut-off of ANI at diagnosis for all-cause mortality was determined to be −0.59. In the comparative and Kaplan–Meier survival analyses, patients with ANI at diagnosis ≥ −0.59 exhibited a significantly higher risk for all-cause mortality and a significantly lower cumulative patient survival rate than those without. Finally, in the multivariable Cox analysis, ANI at diagnosis ≥ −0.59 together with age at diagnosis, was independently associated with all-cause mortality. Therefore, the results of the present study have clinical significance in that it is the first to demonstrate the potential of ANI at diagnosis to estimate all-cause mortality in patients with AAV.

Regarding the mechanism of the potential of the indices indicating NAFLD to estimate all-cause mortality, previous studies provided several hypotheses: both the inflammatory burden of AAV and subsequent insulin resistance may initiate and accelerate NAFLD; NAFLD may be associated with the occurrence of cardiovascular or cerebrovascular diseases; and thus, NAFLD may increase the frequency of all-cause mortality [14,16]. Therefore, we expected ANI favouring ALD compared with NAFLD to be inversely associated with all-cause mortality in patients with AAV. However, the present study demonstrated that the frequency of all-cause mortality significantly increased in proportion to ANI at diagnosis. Based on these results, we inferred the relationship between each of the four parameters comprising a formula of ANI and all-cause mortality in patients with AAV in reverse order.

First, in terms of the male sex, it is an established risk factor for all-cause mortality in the general population [19]. Similarly, in the present study, the all-cause mortality was significantly higher in male than in female patients (19.5% vs. 7.9%, respectively, *p* = 0.006). Additionally, the male patients had a significantly higher ANI at diagnosis than the female patients (1.85 vs. −2.92, *p* < 0.001). In addition, in the univariable Cox analysis, the male sex was significantly associated with all-cause mortality in patients with AAV (HR 2.913, 95% CI 1.441, 5.891, *p* = 0.003). Therefore, it can be concluded that the male sex made a critical contribution to the potential of ANI at diagnosis to estimate all-cause mortality during follow-up in patients with AAV.

Second, in terms of BMI, it has been reported that BMI exhibits a U-shaped association with the rate of all-cause mortality in the general population, in which the lowest risk for all-cause mortality was observed in the range from 22 kg/m^2^ to 25 kg/m^2^ [20,21]. The median BMI in the present study was 22.4 kg/m^2^, which is within the range mentioned above; thus, it can be theoretically assumed that the rate of all-cause mortality can increase as BMI increases or decreases from 22.4 kg/m^2^ in patients with AAV. Therefore, it can be concluded that BMI made a complementary contribution to the potential of ANI at diagnosis to estimate all-cause mortality during follow-up in patients with AAV, regardless of the negative coefficient assigned to BMI.

Third, in terms of the adjusted AST/ALT ratio, no significant difference was found in the median adjusted AST/ALT between deceased and surviving patients. In addition, AST, ALT, and the not-adjusted AST/ALT ratio did not differ between the two groups. Nevertheless, in the univariable Cox analysis, the adjusted AST/ALT ratio tended to be associated with all-cause mortality in patients with AAV (HR 1.725, 95% CI 9.992, 3.001, *p* = 0.053). In addition, previous studies demonstrated that elevated AST/ALT could estimate all-cause mortality [22,23]. Therefore, although the direct involvement of AAV in the liver is rare in real clinical practice [12,24], it can be concluded that the adjusted AST/ALT ratio positively contributed to the potential of ANI at diagnosis to estimate all-cause mortality during follow-up in patients with AAV.

Finally, in terms of MCV, two previous studies reported conflicting results regarding the association between MCV and all-cause mortality: one determined that MCV was proportionally associated with all-cause mortality, whereas the other revealed that it was inversely associated with all-cause mortality in patients under renal replacement therapy [25,26]. In the present study, the deceased patients exhibited a lower median adjusted MCV at diagnosis than the surviving patients (*p* = 0.026). Given the positive coefficient assigned to the adjusted parameter, it can be concluded that the adjusted MCV at diagnosis had an inverse influence on the potential of ANI at diagnosis to estimate all-cause mortality during follow-up in patients with AAV.

In summary, among the four ANI parameters, male sex, and adjusted AST/ALT ratio had a positive potential for estimating all-cause mortality, whereas BMI and MCV had ambivalent and a negative potential for estimating all-cause mortality in patients with AAV. Through this mechanism, it may be concluded that ANI at diagnosis can estimate all-cause mortality during follow-up in AAV patients without significant chronic liver diseases, regardless of the presence of ALD.

Alcohol-related liver disease has been known to account for 30% of the cases of HCC and its related death through the carcinogenic potential of alcohol intake including acetaldehyde toxicity, reactive oxygen species production, impaired immunity, and altered post-translational modifications [27,28]. Based on these concepts, it could be naturally assumed that patients with relatively higher values of ANI at diagnosis in this study might have been exposed to a higher risk of HCC compared with those with lower values of ANI. Therefore, fortunately, there were no patients who had HCC after diagnosis of AAV, but we suggest that efforts to monitor the occurrence of HCC during follow-up in patients with higher values of ANI at diagnosis should be made. A method for monitoring HCC itself or liver nodules with regular ultrasound and using alpha-fetoprotein scores should also be devised [29].

The present study is the first to demonstrate the potential of ANI at diagnosis to estimate all-cause mortality in AAV patients without significant chronic liver diseases and to suggest an additional index for estimating AAV poor outcomes. However, the present study has several limitations. First, above all, due to the retrospective study method, the possibility of uncertain and missing data could not be completely excluded. In addition, due to the retrospective study design, we could not collect “alcohol intake” from the study subjects. However, we believe that this potential critical hurdle was overcome because patients with significant ALD with hepatic dysfunction or alcohol dependence at diagnosis, which were identified by the 10th revised International Classification Diseases-10 and the Korean Drug Utilization Review system, were excluded from this study. Second, the relatively small number of study subjects due to a single-centre study might have limited the external validity and generalisability of the findings, although the AAV cohort of this hospital is the only one including a considerable number of patients with AAV in Korea. Nonetheless, we believe that it has two advantages: Because Korean patients with AAV were enrolled under the same protocol, there were few inter-observer or inter-institutional variations, and the patients could represent the racial and regional characteristics of Korean patients. Third, the area under the curve and the sensitivity and specificity of the cut-off were low. Nevertheless, this study has clinical implications in that, as a pilot study, it suggested methodological procedures to obtain the cut-off value of ANI for estimating all-cause mortality in AAV patients with different ethnic and geographical backgrounds. Fourth, because there is a vicious cycle among multi-step processes including the progress of AAV itself, the adverse effects of strong immunosuppressants according to AAV progression, the occurrence of opportunistic infection, and the progression of AAV as a result of recurrent infection and multi-organ failure, the direct causes of death were not clarified in this study. Fifth, because transient elastography was not performed in all patients at diagnosis, subclinical chronic liver disease could not be excluded from this study more efficiently than ultrasonography or CT scans. Last, the continuous variables are likely to have a real effect on the statistical model rather than the categorical variables; however, this study suggested a categorical variable of ANI at diagnosis ≥ −0.59. This is because we believed that it would be more practical and applicable to use a categorical variable with a cut-off for all-cause mortality in clinical practice despite a slight decrease in statistical reliability. A future study that performs transient elastography, a non-invasive method, on patients newly diagnosed with AAV and prospectively follows them up is expected to overcome these limitations and provide more reliable information.

## 5. Conclusions

The aim of the present study was to investigate whether ANI at diagnosis might be associated with all-cause mortality during follow-up in AAV patients without significant chronic liver diseases. To our knowledge, it was the first to demonstrate the potential of ANI at diagnosis to estimate all-cause mortality during follow-up in patients with AAV, regardless of the presence of ALD.

## Figures and Tables

**Table 1:** Characteristics of patients (N = 270).

Variables	Values
*At diagnosis*	
Demographic data	
Age (years)	61.0 (50.0–69.3)
Male sex (N, (%))	93 (34.4)
Female sex (N, (%))	177 (66.6)
BMI (kg/m^2^)	22.4 (20.2–24.5)
Smoking history (N, (%))	9 (3.3)
AAV Subtypes (N, (%))	
MPA	152 (56.3)
GPA	66 (24.4)
EGPA	52 (19.3)
ANCA positivity (N, (%))	
MPO-ANCA (or P-ANCA) positivity	191 (70.7)
PR3-ANCA (or C-ANCA) positivity	41 (15.2)
AAV-specific indices	
BVAS	12.0 (7.0–18.0)
FFS	1.0 (0–2.0)
Acute-phase proteins	
ESR (mm/h)	60.5 (22.0–96.0)
CRP (mg/L)	13.8 (1.6–68.9)
Comorbidities at diagnosis (N, (%))	
Type 2 diabetes mellitus	72 (26.7)
Arterial hypertension	110 (40.7)
Hyperlipidaemia	55 (20.4)
ANI-related variables	
MCV (fL)	90.4 (87.5–93.9)
Adjusted MCV * (fL)	92.0 (92.0–93.9)
AST (IU/L)	18.0 (15.0–24.0)
ALT (IU/L)	16.0 (11.0–25.0)
AST/ALT	1.2 (0.9–1.6)
Adjusted AST/ALT **	1.2 (0.9–1.6)
BMI (kg/m^2^)	22.4 (20.2–24.5)
Male sex	93 (34.4)
ANI	−1.15 (−3.89–1.55)
*During follow-up*	
All-cause mortality	
All-cause mortality (N, (%))	32 (11.9)
Follow-up duration based on all-cause mortality (months)	47.8 (15.7–82.1)
Medications administered during follow-up (N, (%))	
Glucocorticoid	254 (94.1)
Cyclophosphamide	153 (56.7)
Rituximab	45 (16.7)
Azathioprine	146 (54.1)
Mycophenolate mofetil	51 (18.9)
Tacrolimus	23 (8.5)
Methotrexate	23 (8.5)

Values are expressed as a median (25~75 percentiles) or a number (percentage). * Adjusted MCV: The range of MCV as a variable for ALI is 92 to 103. Therefore, MCV below 92 is set at 92, and MCV above 103 is set at 103. ** Adjusted AST/ALT: The range of AST/ALT as a parameter of ALI cannot exceed 3. Therefore, AST/ALT above 3 is set at 3. AAV: ANCA-associated vasculitis; ANCA: antineutrophil cytoplasmic antibody; BMI: body mass index; MPA: microscopic polyangiitis; GPA: granulomatosis with polyangiitis; EGPA: eosinophilic GPA; MPO: myeloperoxidase; P: perinuclear; PR3: proteinase 3; C: cytoplasmic; BVAS: Birmingham Vasculitis Activity Score; FFS: Five-Factor Score; ESR: erythrocyte sedimentation rate; CRP: C-reactive protein; ANI: the alcoholic liver disease/nonalcoholic fatty liver disease index; MCV: mean corpuscular volume; AST: aspartate aminotransferase; ALT: alanine aminotransferase.

**Table 2:** Cox hazards model analysis of variables at diagnosis for all-cause mortality during follow-up in patients with AAV.

Variables	Univariable			Multivariable		
	HR	95% CI	*p*-Value	HR	95% CI	*p*-Value
Age	1.089	1.048, 1.133	<0.001	1.061	1.017, 1.107	0.006
Male sex *	2.913	1.441, 5.891	0.003			
BMI *	1.114	1.015, 1.223	0.024			
Smoking history	1.902	0.453, 7.980	0.380			
MPO-ANCA (or P-ANCA) positivity	2.0117	0.827, 4.918	0.123			
PR3-ANCA (or C-ANCA) positivity	0.170	0.023, 1.248	0.082			
BVAS	1.086	1.037, 1.138	<0.001	1.051	0.993, 1.112	0.088
FFS	2.019	1.444, 2.823	<0.001	1.286	0.844, 1.959	0.242
ESR	1.012	1.003, 1.022	0.010	1.000	0.988, 1.012	0.962
CRP	1.009	1.004, 1.014	<0.001	1.004	0.998, 1.011	0.214
Type 2 diabetes mellitus	1.090	0.514, 2.311	0.822			
Arterial hypertension	1.374	0.686, 2.752	0.370			
Hyperlipidaemia	1.761	0.833, 3.723	0.139			
ANI ≥ −0.59	2.550	1.228, 5.296	0.012	2.479	1.149, 5.349	0.021

*: Because male sex and BMI are the parameters of the equation for ANI, they were not included in the multivariable Cox analysis. AAV: ANCA-associated vasculitis; ANCA: antineutrophil cytoplasmic antibody; HR: hazard ratio; CI: confidence interval; BMI: body mass index; MPO: myeloperoxidase; P: perinuclear; PR3: proteinase 3; C: cytoplasmic; BVAS: Birmingham Vasculitis Activity Score; ESR: erythrocyte sedimentation rate; CRP: C-reactive protein; ANI: the alcoholic liver disease/nonalcoholic fatty liver disease index.

## Data Availability

The dataset collected and/or analysed in the present study is avail-able upon request from the corresponding author.

## References

[1] Seitz H.K., Bataller R., Cortez-Pinto H., Gao B., Gual A., Lackner C., Mathurin P., Mueller S., Szabo G., Tsukamoto H. (2018). Alcoholic liver disease. Nat. Rev. Dis. Primers.

[2] Cotter T.G., Rinella M. (2020). Nonalcoholic Fatty Liver Disease 2020: The State of the Disease. Gastroenterology.

[3] Li B., Zhang C., Zhan Y.T. (2018). Nonalcoholic Fatty Liver Disease Cirrhosis: A Review of Its Epidemiology, Risk Factors, Clinical Presentation, Diagnosis, Management, and Prognosis. Can. J. Gastroenterol. Hepatol..

[4] Idalsoaga F., Kulkarni A.V., Mousa O.Y., Arrese M., Arab J.P. (2020). Non-alcoholic Fatty Liver Disease and Alcohol-Related Liver Disease: Two Intertwined Entities. Front. Med..

[5] Dunn W., Angulo P., Sanderson S., Jamil L.H., Stadheim L., Rosen C., Malinchoc M., Kamath P.S., Shah V.H. (2006). Utility of a new model to diagnose an alcohol basis for steatohepatitis. Gastroenterology.

[6] Jennette J.C., Falk R.J., Bacon P.A., Basu N., Cid M.C., Ferrario F., Flores-Suarez L.F., Gross W.L., Guillevin L., Hagen E.C. (2013). 2012 revised International Chapel Hill Consensus Conference Nomenclature of Vasculitides. Arthritis Rheum..

[7] Watts R., Lane S., Hanslik T., Hauser T., Hellmich B., Koldingsnes W., Mahr A., Segelmark M., Cohen-Tervaert J.W., Scott D. (2007). Development and validation of a consensus methodology for the classification of the ANCA-associated vasculitides and polyarteritis nodosa for epidemiological studies. Ann. Rheum. Dis..

[8] Suppiah R., Robson J.C., Grayson P.C., Ponte C., Craven A., Khalid S., Judge A., Hutchings A., Merkel P., Luqmani R. (2022). 2022 American College of Rheumatology/European Alliance of Associations for Rheumatology classification criteria for microscopic polyangiitis. Ann. Rheum. Dis..

[9] Robson J.C., Grayson P.C., Ponte C., Suppiah R., Craven A., Judge A., Khalid S., Hutchings A., Watts R.A., Merkel P.A. (2022). 2022 American College of Rheumatology/European Alliance of Associations for Rheumatology classification criteria for granulomatosis with polyangiitis. Ann. Rheum. Dis..

[10] Grayson P.C., Ponte C., Suppiah R., Robson J.C., Craven A., Judge A., Khalid S., Hutchings A., Luqmani R.A., Watts R.A. (2022). 2022 American College of Rheumatology/European Alliance of Associations for Rheumatology Classification Criteria for Eosinophilic Granulomatosis with Polyangiitis. Ann. Rheum. Dis..

[11] Yazici H., Tascilar K., Yazici Y. (2023). 2022 American College of Rheumatology/European Alliance of Associations for Rheumatology classification criteria sets for three types of antineutrophilic cytoplasmic antibody-associated vasculitis. Curr. Opin. Rheumatol..

[12] Mukhtyar C., Lee R., Brown D., Carruthers D., Dasgupta B., Dubey S., Flossmann O., Hall C., Hollywood J., Jayne D. (2009). Modification and validation of the Birmingham Vasculitis Activity Score (version 3). Ann. Rheum. Dis..

[13] Guillevin L., Pagnoux C., Seror R., Mahr A., Mouthon L., Toumelin P.L., French Vasculitis Study Group (2011). The Five-Factor Score revisited: Assessment of prognoses of systemic necrotizing vasculitides based on the French Vasculitis Study Group (FVSG) cohort. Medicine.

[14] Park P.G., Pyo J.Y., Ahn S.S., Choi H.J., Song J.J., Park Y.B., Huh J.H., Lee S.-W. (2022). Fatty Liver Index Independently Predicts All-Cause Mortality in Patients with Antineutrophil Cytoplasmic Antibody-Associated Vasculitis but No Substantial Liver Disease. Front. Cardiovasc. Med..

[15] Park P.G., Pyo J.Y., Ahn S.S., Song J.J., Park Y.B., Huh J.H., Lee S.-W. (2023). New index using triglyceride glucose-body mass index for predicting mortality in patients with antineutrophil cytoplasmic antibody-associated vasculitis. Front. Med..

[16] Whang J.Y., Park P.G., Park Y.B., Huh J.H., Lee S.W. (2023). Non-alcoholic fatty liver disease fibrosis score is a useful index for predicting all-cause mortality in patients with antineutrophil cytoplasmic antibody-associated vasculitis. Front. Med..

[17] Willeke P., Schlüter B., Limani A., Becker H., Schotte H. (2016). Liver involvement in ANCA-associated vasculitis. Clin. Rheumatol..

[18] Zhu Q., Li F., Xie X., Chen B., Yu Q., Wei Y., Ge Y. (2022). Relationship Between Gender and 1-Year Mortality in ANCA-Associated Vasculitis Patients: A Single-Center Retrospective Analysis and Meta-Analysis. Front. Med..

[19] Murray C.J., Atkinson C., Bhalla K., Birbeck G., Burstein R., Chou D., Chugh S.S., Cohen A., Colson K.E., Cooper L.T. (2013). The state of US health, 1990–2010: Burden of diseases, injuries, and risk factors. JAMA.

[20] Kim N.H., Lee J., Kim T.J., Kim N.H., Choi K.M., Baik S.H., Choi D.S., Pop-Busui R., Park Y., Kim S.G. (2015). Body Mass Index and Mortality in the General Population and in Subjects with Chronic Disease in Korea: A Nationwide Cohort Study (2002–2010). PLoS ONE.

[21] Aune D., Sen A., Prasad M., Norat T., Janszky I., Tonstad S., Romundstad P., Vatten L.J. (2016). BMI and all cause mortality: Systematic review and non-linear dose-response meta-analysis of 230 cohort studies with 3.74 million deaths among 30.3 million participants. BMJ.

[22] Liu X., Liu P. (2022). Elevated AST/ALT ratio is associated with all-cause mortality in patients with stable coronary artery disease: A secondary analysis based on a retrospective cohort study. Sci. Rep..

[23] Liu H., Ding C., Hu L., Li M., Zhou W., Wang T., Zhu L., Bao H., Cheng X. (2021). The association between AST/ALT ratio and all-cause and cardiovascular mortality in patients with hypertension. Medicine.

[24] Choi C.B., Park Y.B., Lee S.W. (2019). Antineutrophil Cytoplasmic Antibody-Associated Vasculitis in Korea: A Narrative Review. Yonsei Med. J..

[25] Hsieh Y.P., Chang C.C., Kor C.T., Yang Y., Wen Y.K., Chiu P.F. (2017). Mean Corpuscular Volume and Mortality in Patients with CKD. Clin. J. Am. Soc. Nephrol..

[26] Honda H., Kimachi M., Kurita N., Joki N., Nangaku M. (2020). Low rather than high mean corpuscular volume is associated with mortality in Japanese patients under hemodialysis. Sci. Rep..

[27] Ganne-Carrié N., Nahon P. (2019). Hepatocellular carcinoma in the setting of alcohol-related liver disease. J. Hepatol..

[28] Testino G., Leone S., Borro P. (2014). Alcohol and hepatocellular carcinoma: A review and a point of view. World J. Gastroenterol..

[29] Mazzotta A.D., Pascale A., Cano L., Rosmorduc O., Allard M., Cunha A.S., Adam R., Cherqui D., Vibert E., Golse N. (2021). Number of hepatocellular carcinoma nodules in patients listed for liver transplantation within alpha-fetoprotein score: A new prognostic risk factor. Transpl. Int..

